# Habilidades funcionales de niños, niñas y adolescentes con parálisis cerebral y su relación con el compromiso motor y la discapacidad intelectual en Argentina

**DOI:** 10.31053/1853.0605.v80.n4.40834

**Published:** 2023-12-26

**Authors:** Mercedes Ruiz Brunner, L. Johana Escobar Zuluaga, E. Federico Sánchez, M. Elisabeth Cieri, Ana Laura Condinanzi, Natalia Herrera Sterren, Ana Carolina Zinni, Marcelo F. Barilla, Mailen Araceli Cernadas, Eduardo Cuestas

**Affiliations:** 1 Universidad Nacional de Córdoba. Facultad de Ciencias Médicas. Instituto de Investigaciones Clínicas y Epidemiológicas (INICyE) Córdoba Argentina; 3 Centro de Investigaciones y Estudios sobre Cultura y Sociedad (CIECS), CONICET/UNC Córdoba Argentina; 4 Asociación en Defensa del Infante Neurológico (AEDIN) Ciudad Autónoma de Buenos Aires Argentina; 5 Sub Secretaría de Discapacidad, Rehabilitación e Inclusión, Ministerio de Salud Córdoba Argentina; 6 Servicio Integral de Rehabilitación y Educación Terapéutica (SIRET) Reconquista Sata Fe Argentina; 7 Departamento de Pediatría y Neonatología, Hospital Privado Universitario de Córdoba Córdoba Argentina

**Keywords:** parálisis cerebral, niño, discapacidad intelectual, desarrollo infantil, habilidades motoras, cerebral palsy, child, intellectual disability, Child development, motor skills, paralisia cerebral, criança, deficiência intelectual, desenvolvimento infantil, habilidades motoras

## Abstract

El compromiso motor caracteriza la parálisis cerebral (PC), y suele asociarse a la discapacidad intelectual (DI). Se han desarrollado sistemas de clasificación estandarizados para describir las funciones de personas con PC.

**Objetivo:**

caracterizar funcionalmente a niños, niñas y adolescentes de 0 a 18 años con PC de Argentina e indagar la asociación entre el compromiso motor (GMFCS), la DI y las clasificaciones funcionales.

**Métodos:**

Estudio transversal. Se recolectaron datos a través de entrevistas a las familias y revisión de historias clínicas. Se incluyeron personas con PC. Los datos se recolectaron de 19 instituciones de distintas ciudades de Argentina. Para el análisis de los datos se utilizó test de Fisher y odds ratio [IC95%], con significación <0,05.

**Resultados:**

participaron 182 niños, niñas y adolescentes con PC. Según clasificación GMFCS prevaleció el nivel V con 36,3%. Quienes presentan compromiso motor más severo (GMFCS IV-V), tienen 72 [25,4;206,0] veces y 13 [5,9;28,2] veces más probabilidad de presentar un nivel severo de MACS y CFCS respectivamente. Presentaron 34 [7,9;146,0] más probabilidad de tener un nivel severo de EDACS. Quienes presentaron DI tuvieron 10 [5,1;20,5] veces más probabilidad de presentar un nivel severo GMFCS, 6 [3,4;13,2] veces más un nivel severo MACS, 4 [2,0;7,8] veces más nivel severo CFCS, y 4 [1,9;9,5] veces más de presentar nivel severo de EDACS.

**Conclusión:**

el nivel de GMFCS y la presencia de DI influyen en la funcionalidad general y aumentan la severidad en el compromiso, habilidades manuales, de comunicación y para comer y beber.

## Abstract (en)

**Introduction:**

Motor compromise characterizes cerebral palsy (CP), and is often associated with intellectual disability (ID). Standardized classification systems have been developed to describe the functions of people with CP. The aim was to functionally characterize children and adolescents aged 0 to 18 years with CP in Argentina and to investigate the association between motor compromise (GMFCS), ID and functional classifications.

**Methods:**

Cross-sectional study. Data were collected through family interviews and medical records review. Individuals with CP were included. Data were collected from 19 institutions in different cities of Argentina. Fisher's test and odds ratio [IC95%] were used for data analysis, with significance <0.05.

**Results:**

182 children and adolescents with CP participated. According to GMFCS classification, level V prevailed with 36.3%. Those with more severe motor compromise (GMFCS IV-V) were 72 [25.4;206.0] times and 13 [5.9;28.2] times more likely to present a severe level of MACS and CFCS, respectively. They were 34 [7.9;146.0] times more likely to have a severe level of EDACS. Those with ID were 10 [5.1;20.5] times more likely to have severe GMFCS, 6 [3.4;13.2] times more likely to have severe MACS, 4 [2.0;7.8] times more likely to have severe CFCS, and 4 [1.9;9.5] times more likely to have severe EDACS.

**Conclusion:**

the level of GMFCS and the presence of DI influence general functionality and increase severity in engagement, manual, communication, eating and drinking skills.

## Resumo

**Introdução:**

O envolvimento motor caracteriza a paralisia cerebral (PC), e está frequentemente associado à deficiência intelectual (DI). Foram desenvolvidos sistemas de classificação normalizados para descrever as funções das pessoas com PC.

**Objectivo:**

Caracterizar funcionalmente crianças e adolescentes de 0-18 anos com PC na Argentina e investigar a associação entre deficiência motora (GMFCS), DI e classificações funcionais.

**Materiais e Métodos:**

Estudo transversal. Os dados foram recolhidos através de entrevistas familiares e revisão de registos médicos. Foram incluídas pessoas com PC. Os dados foram recolhidos de 19 instituições em diferentes cidades da Argentina. Teste de Fisher e odds ratio [CI95%] foram utilizados para análise de dados, com significado <0,05.

**Resultados:**

Participaram 182 crianças e adolescentes com PC. De acordo com a classificação do GMFCS, o nível V prevaleceu com 36,3%. Aqueles com comprometimento motor mais grave (GMFCS IV-V) apresentaram 72 [25,4;206,0] vezes e 13 [5,9;28,2] vezes mais chances de ter MACS e CFCS graves, respetivamente. Tinham 34 [7,9;146,0] vezes mais probabilidades de ter um nível grave de EDACS. As pessoas com DI tinham 10 [5,1;20,5] vezes mais probabilidades de ter GMFCS grave, 6 [3,4;13,2] vezes mais probabilidades de ter MACS grave, 4 [2,0;7,8] vezes mais probabilidades de ter CFCS grave e 4 [1,9;9,5] vezes mais probabilidades de ter EDACS grave.

**Conclusão:**

O nível de GMFCS e a presença de DI influenciam o funcionamento geral e aumentam a gravidade das capacidades de envolvimento, manuais, de comunicação, de comer e de beber.

**CONCEPTOS CLAVE**

Qué se sabe sobre el tema: En las personas con parálisis cerebral (PC) prevalece el compromiso motor y muchas veces se encuentra asociada la discapacidad intelectual (DI).

Las funciones de niños, niñas y adolescentes con PC se pueden clasificar según el sistema de clasificación de la función motora gruesa (GMFCS), sistema de clasificación de las habilidades manuales (MACS), sistema de clasificación de las funciones de la comunicación (CFCS), sistema de clasificación de las habilidades para comer y beber (EDACS).

Qué aporta este trabajo: A medida que aumenta el compromiso motor grueso aumentan las probabilidades de presentar compromiso severo en las habilidades manuales, de comunicación y para comer y beber en niños, niñas y adolescentes con PC.

Cuando hay presencia de DI en niños, niñas y adolescentes con PC aumentan las probabilidades de presentar compromiso severo en las funciones motoras gruesas, las habilidades manuales, de comunicación y para comer y beber.

**Divulgación**

La parálisis cerebral (PC) es la discapacidad motora más frecuente en pediatría y se presenta de forma muy variada en cada niño. Para describir cómo se presenta la PC existen clasificaciones funcionales. En este trabajo observamos que mientras mayor sea el compromiso motor y también cuando existe discapacidad intelectual, aumenta la severidad en el compromiso de las habilidades manuales, para comunicarse y para comer y beber.

## Introducción

La parálisis cerebral (PC) se define como un grupo de trastornos permanentes del desarrollo del movimiento y la postura, consecuente a una alteración no progresiva del cerebro en desarrollo ^1^ . La versatilidad con la que se manifiesta en cada persona complejizó, por varios años, el poder establecer una única definición ^2^ . Más allá del diagnóstico, es central para poder plantear los procesos de rehabilitación conocer las funciones que cada persona es capaz de realizar.

El compromiso motor es una característica esencial en la PC, es por eso que en 1997 se desarrolla el primer sistema de clasificación de las funciones motoras gruesas (Gross Motor Function Clasificaction System – GMFCS), conocido como patrón de oro para la descripción funcional de la PC ^3^ . A nivel internacional se han desarrollado sistemas de clasificación estandarizados para describir las funciones de niños, niñas y adolescentes con PC de forma sistemática y validada ^4^ . Los sistemas de clasificación funcional se clasifican en cinco niveles, discriminando variaciones significativas entre expresiones o etapas de progresión de una condición en salud ^5^ .

Una de las condiciones asociadas a la PC es la discapacidad intelectual. Estudios plantean que existiría una correlación entre la presencia de discapacidad intelectual con mayores limitaciones funcionales ^6^ . Se desconoce si esta asociación se observa también en nuestro medio.

Este estudio tuvo por objetivo caracterizar funcionalmente a niños, niñas y adolescentes con parálisis cerebral de Argentina indagando la asociación entre el compromiso motor, la discapacidad intelectual y las clasificaciones funcionales de habilidades manuales, comunicación y habilidades para comer y beber.

## Materiales y Métodos

Se realizó un estudio transversal con recolección de datos de manera prospectiva. La muestra estuvo constituida por niños, niñas y adolescentes con PC de entre 2 y 18 años de edad, que asistían en forma ambulatoria a diferentes instituciones. Se incluyeron 19 centros de rehabilitación y centros educativos terapéuticos de quince ciudades de Argentina (Reconquista, CABA, Córdoba, Jujuy, Catamarca, Unquillo, Villa Dolores, Cruz del Eje, Río Ceballos, Villa Maria, Río IV, La Plata, Pacheco, Mendoza y Rosario).

Los datos fueron recolectados entre octubre de 2021 y octubre de 2022. Se realizó un muestreo secuencial incluyendo el mayor número de casos en cada institución. Se incluyeron niños, niñas y adolescentes con diagnóstico confirmado de PC. Se adoptó la definición de caso de PC utilizada en la vigilancia de la PC en Europa (SCPE), el Registro de PC de Australia y el Registro de PC de Bangladesh, que permite el uso de cualquier definición de PC que incluya los siguientes cinco elementos clave comunes a las definiciones publicadas ^1, 7^ .

Definición de caso de la parálisis cerebral:

- Es un término genérico para un grupo de trastornos.
- Es una condición que es permanente pero no inmutable.
- Implica un trastorno del movimiento y / o postura y de la función motora.
- Se debe a una interferencia, lesión o anomalía no progresiva.
- La interferencia, lesión o anomalía se origina en el cerebro inmaduro.

Se excluyeron aquellos niños, niñas y adolescentes que no asistieron a la institución durante el proceso de recolección de datos, o que no aceptaron participar en el estudio. Las familias que aceptaron participar firmaron un consentimiento informado.

Para la recolección de los datos clínicos y sociodemográficos, se utilizaron dos instrumentos, el Perfil de funcionamiento en niños, niñas y adolescentes con PC y el formulario del Registro Argentino de PC (RAR-PC). Los datos se obtuvieron a través de un cuestionario semiestructurado dirigido a los cuidadores sobre información familiar y los niveles de las clasificaciones funcionales fueron clasificados por médicos y fisioterapeutas de acuerdo con los lineamientos de cada sistema de clasificación. La evaluación se realizó durante los exámenes físicos y los procesos de rehabilitación, corroborando los datos con las historias clínicas.

Las variables clínicas y sociodemográficas evaluadas fueron las siguientes: edad, nivel educativo de la madre, provincia, si cuenta con seguridad social y certificado único de discapacidad (CUD), Clasificación Europea de la PC (SCPE), condiciones asociadas y cuatro clasificaciones funcionales de la PC: Sistema de clasificación de la función motora gruesa (GMFCS), sistema de clasificación de las habilidades manuales (MACS), sistema de clasificación de las funciones de la comunicación (CFCS), sistema de clasificación de las habilidades para comer y beber (EDACS). Las descripciones de los niveles de estas clasificaciones se encuentran descritas en la Tabla 1.

**Tabla N°1:** Niveles de funcionalidad de los sistemas de clasificación para niños, niñas y adolescentes con parálisis cerebral

Sistemas de Clasificación	Nivel I	Nivel II	Nivel III	Nivel IV	Nivel V
GMFCS	Anda sin limitaciones.	Anda con limitaciones.	Anda utilizando un dispositivo de movilidad con sujeción manual en la mayoría de los espacios interiores.	Usan métodos de movilidad que requieren asistencia física o movilidad motorizada en la mayoría de los entornos.	Transportado en silla de ruedas en todos los entornos.
MACS	Manipula objetos fácil y exitosamente	Manipula la mayoría de los objetos, pero con un poco de reducción en la calidad y/o velocidad del logro.	Manipula los objetos con dificultad; necesita ayuda para preparar. y/o modificar actividades.	Manipula una limitada selección de objetos fácilmente manipulables en situaciones adaptadas.	No manipula objetos y tiene habilidad severamente limitada para ejecutar aún acciones sencillas.
CFCS	Emisor eficaz y receptor eficaz con interlocutores conocidos y desconocidos	Emisor y/o Receptor Eficaz, pero con un ritmo más lento con interlocutores conocidos y/o desconocidos.	Emisor Eficaz y Receptor Eficaz con los interlocutores conocidos.	Emisor y/o Receptor Inconstante con los interlocutores conocidos.	Emisor y Receptor Raramente Eficaz aun con interlocutores conocidos.
						EDACS	Come y bebe con seguridad y eficiencia.	Come y bebe con seguridad, pero con algunas limitaciones en la eficiencia.	Come y bebe con algunas limitaciones en la seguridad; puede tener algunas limitaciones en la eficiencia.	Come y bebe con limitaciones significativas de seguridad.	Es incapaz de comer y beber con seguridad – la alimentación por sonda puede ser considerada para proporcionar la nutrición.

GMFCS: Sistema de clasificación de la función motora gruesa. MACS: Sistema de clasificación de las habilidades manuales. CFCS: Sistema de clasificación de las funciones de la comunicación. EDACS: Sistema de clasificación de las habilidades para comer y beber.

### Análisis Estadísticos

Para el análisis de los datos, las variables categóricas se describieron en valores absolutos y relativos con sus respectivos IC 95%. Las variables continuas normales se describieron con media ± desviación estándar. Para determinar la asociación entre el compromiso motor y la discapacidad intelectual y las clasificaciones funcionales de habilidades manuales, comunicación y habilidades para comer y beber, se realizó un test de Fisher. Teniendo en cuenta las variables que mostraron significación en el análisis bivariado, se calcularon las odds ratio con un IC del 95%. La significación estadística se estableció en p < 0,05. Los análisis estadísticos se realizaron con los programas estadísticos MedCalc 18.2.1 (MedCal Software Ltd, Bélgica).

### Consideraciones éticas

Este estudio fue aprobado por el comité de ética del Hospital Nacional de Clínicas de Córdoba. Se obtuvieron los consentimientos informados por escrito de los niños y niñas mayores de 13 años que pudieran hacerlo, padres, tutores y/o cuidadores y el asentimiento de los niños y niñas.

## Resultados

Se recolectaron datos de 182 niños, niñas y adolescentes con parálisis cerebral de 0 a 18 años. La edad promedio fue de 8,4 ± 4,7 años con un mínimo de 5 meses y un máximo de 18 años, y con prevalencia del sexo masculino. El nivel educativo de la madre fue mayormente el terciario o universitaria, seguido de quienes presentaban secundario completo o universitaria incompleta, muy pocos tenían estudios primarios incompletos. La mayor densidad de población donde se realizó el registro fue en las provincias de Buenos Aires y Córdoba (Tabla 2).

**Tabla N° 2:** Caracterización demográfica de la población (n=182)

	n	%	IC95%
Sexo			
Femenino	74	40,7%	(33,4; 48,2)
Masculino	108	59,3%	(51,7; 66,0)
Nivel educativo de la madre			
Sin estudios o primaria incompleta	1	0,6%	(0,0; 3,1)
Primaria Completa o Secundaria Incompleta	48	26,4%	(20,1; 33,4)
Secundaria Completa o Terciario / Universitario Incompleta	60	33,0%	(26,2; 40,3)
Terciario / Universitario Completo o Post grado	67	36,8%	(29,8; 44,2)
Sin dato	6	3,3%	(1,2; 7,0)
Provincia donde vive			
Buenos Aires	65	35,7%	(28,7; 43,1)
CABA	14	7,7%	(4,2; 12,5)
Catamarca	1	0,5%	(0.0; 2,9)
Córdoba	47	25,8%	(19,6; 32,7)
Jujuy	15	8,2%	(4,6; 13,1)
Mendoza	6	3,3%	(1,2; 7,0)
Rio Negro	1	0,5%	(0,0; 2,9)
Santa Fe	33	18,1%	(12,7; 24,4)
Cuenta con obra social (si)	144	79,1%	(72,5; 87,8)
Cuenta con Certificado único de discapacidad CUD (si)	177	97,3%	(93,8; 99,1)
Recibe terapias de rehabilitación (si)	178	97,8%	(97,5; 99,4)

Según el tipo de PC se encontró en mayor proporción la PC Espástica tanto bilateral como unilateral. Entre las condiciones asociadas, se observó en un mayor número de casos la discapacidad intelectual aislada o junto a epilepsia (Tabla 3).

**Tabla N° 3:** Caracterización clínica del tipo de parálisis cerebral y condiciones asociadas (n=182)

	n	%	IC95%
Clasificación según SCPE			
PC Espástica Unilateral	49	26,9%	(20,6; 34,0)
PC Espástica Bilateral	**116**	**63,7%**	**(56,3; 70,7)**
PC Atáxica	4	2,2%	(0,6; 5,5)
PC Discinética	7	3,9%	(1,5; 7,8)
PC Distónica	6	3,3%	(1,2; 7,0)
Condiciones asociadas			
Epilepsia	15	8,2%	(4,6; 13,2)
Epilepsia y Disc. Intelectual	**54**	**29,7%**	**(23,2; 36,9)**
Disc. Intelectual	50	27,5%	(21,5; 34,6)
No presenta	53	29,1%	(22,6; 36,3)
No especifica (sin dato)	10	5,5%	(2,7; 9,9)

En negrita se señalan aquellos datos con mayores prevalencias.

De acuerdo a los datos recolectados de las clasificaciones funcionales, tanto en la clasificación de la función motora gruesa (GMFCS), de las habilidades manuales (MACS) y de comunicación (CFCS) se observó una mayor prevalencia en el nivel V que corresponde al nivel de mayor severidad. Solo en la clasificación de las habilidades para comer y beber (EDACS) se observó mayor prevalencia en el nivel I correspondiente a un compromiso leve (Tabla 4).

**Tabla N°4:** Caracterización funcional de la población de niños, niñas y adolescentes con parálisis cerebral (n=182)

Sistema de clasificación	Nivel de compromiso	n	%	IC95%
GMFCS	Nivel I	32	17,6%	(25,2; 39,3)
	Nivel II	21	11,5%	(7,2; 17,0)
	Nivel III	29	15,9%	(10,9; 22,0)
	Nivel IV	34	18,7%	(13,3; 25,1)
	**Nivel V**	**66**	**36,3%**	(29,3; 43,7)
MACS	Nivel I	35	19,2%	(13,7; 25,6)
	Nivel II	36	19,8%	(14,2; 26,3)
	Nivel III	23	12,6%	(8,1; 18,3)
	Nivel IV	39	21,4%	(15,6; 28,0)
	**Nivel V**	**46**	**25,3%**	(19,1; 32,2)
	Sin dato	3	1,6%	(0,32; 4,6)
CFCS	Nivel I	51	28,0%	(21,6; 35,1)
	Nivel II	19	10,4%	(6,3; 15,7)
	Nivel III	37	20,3%	(14,7; 26,8)
	Nivel IV	27	14,8%	(9,9; 20,8)
	**Nivel V**	**46**	**25,3%**	(19,1; 32,2)
	Sin dato	2	1,1%	(0,1; 3,9)
EDACS	Nivel I	65	35,7%	(28,7; 43,1)
	Nivel II	30	16,5%	(11,4; 22,7)
	**Nivel III**	**38**	**20,9%**	(15,2; 27,5)
	Nivel IV	19	10,4%	(6,3; 15,7)
	Nivel V	28	15,4%	(10,4; 21,4)
	Sin dato	2	1,1%	(0,1; 3,9)

En negrita se señalan aquellos datos con mayores prevalencias.

GMFCS: Sistema de clasificación de la función motora gruesa. MACS: Sistema de clasificación de las habilidades manuales. CFCS: Sistema de clasificación de las funciones de la comunicación. EDACS: Sistema de clasificación de las habilidades para comer y beber.

En la tabla 5, se comparó el nivel del compromiso motor grueso GMFCS con las otras clasificaciones funcionales. Se observó relación estadísticamente significativa con las habilidades manuales MACS (<0,0001), con las habilidades de comunicación CFCS (<0,0001), y con las habilidades para comer y beber EDACS (<0,0001). Se observó que los niños, niñas y adolescentes que presentaron GMFCS un compromiso motor severo, es decir nivel IV-V, tuvieron 72 veces más probabilidad de presentar un nivel severo de MACS y 13 veces más de presentar un nivel severo de CFCS. Respecto a las habilidades para comer y beber, presentaron 34 veces más de presentar un nivel severo de EDACS (Tabla 5).

**Tabla N° 5:** Asociación entre nivel de compromiso motor y otras clasificaciones funcionales

	GMFCS I a III			GMFCS IV y V					
Variable	n	%	IC95%	n	%	IC95%	p	OR [IC95%]	p del OR
MACS									

Nivel I a III (n= 94)	77	81,9	[72,6; 89,1]	17	18,1	[10,9; 27,4]	**< 0,0001**	72,4 [25,4;206,0]	**< 0,0001**
Nivel IV y V(n= 85)	5	5,9	[2,0;13,2]	**80**	**94,1**	[86,8; 98,0]			
CFCS									
Nivel I a III(n= 107)	72	67,3	[57,5; 76,1]	35	32,7	[23,9; 42,4]	**< 0,0001**	13 [5,9; 28,2]	**< 0,0001**
Nivel IV y V(n=73)	10	13,7	[6,8; 23,7]	**63**	**86,3**	[76,2; 93,2]			
EDACS									
Nivel I a III(n=133)	80	60,2	[51,3; 68,6]	53	39,8	[31,4; 48,6]	**<0,0001**		
Nivel IV y V	2	4,3	[0,5; 14,6]	**45**	**95,7**	[85,4; 99,5]		34,0 [7,9; 146,0]	**< 0,0001**
(n=47)									

Los valores en negrita corresponden a valores de p estadísticamente significativos

GMFCS: Sistema de clasificación de la función motora gruesa. MACS: Sistema de clasificación de las habilidades manuales. CFCS: Sistema de clasificación de las funciones de la comunicación. EDACS: Sistema de clasificación de las habilidades para comer y beber. Nivel I a III corresponden a compromisos leves y moderados, Nivel IV y V corresponden a compromisos severos.

Se analizó también la relación entre la presencia de discapacidad intelectual con las clasificaciones funcionales y se observaron relaciones estadísticamente significativas con el nivel de compromiso motor GMFCS (< 0,0001), el nivel de habilidades manuales MACS (< 0,0001), el nivel de comunicación CFCS (< 0,0001) y nivel de habilidades para comer y beber EDCS (<0,0002). Los niños, niñas y adolescentes con PC que presentaron discapacidad intelectual tuvieron 10 veces más probabilidad de presentar un nivel severo de GMFCS, 6 veces más de presentar un nivel severo de MACS y 4 veces más de presentar un nivel severo de CFCS. La severidad de las habilidades para comer y beber presentó una relación con la discapacidad intelectual, por lo que quienes presentaron discapacidad intelectual tienen 4 veces más probabilidades de presentar un nivel severo de EDACS (Tabla 6).

**Tabla N° 6:** Asociación entre la discapacidad intelectual y las clasificaciones funcionales

	Sin Discapacidad Intelectual			Con Discapacidad Intelectual			p	OR [IC95%]	p del OR
Variable	n	%	IC95%	n	%	IC95%			
GMFCS									
Nivel I a III(n=82)	58	70,7	[59,6; 80,2]	24	29,3	[19,8; 40,4]	**< 0,0001**	10 [5,1; 20,5]	**< 0,0001**
Nivel IV y V(n=100)	19	19,0	[11,8; 28,1]	**81**	**81,0**	[71,9; 88,2]			
MACS									
Nivel I a III(n= 94)	59	62,8	[52,2; 72,5]	35	37,2	[27,5; 47,8]	**< 0,0001**	6,7 [3,4;13,2]	**< 0,0001**
Nivel IV y V(n=85)	17	20,0	[12,1; 30,1]	**68**	**80,0**	[69,9; 87,9]			
CFCS									
Nivel I a III(n= 107)	59	55,1	[45,2; 64,7]	48	44,9	[35,3; 54,8]	**< 0,0001**	4,0 [2,0; 7,8]	**< 0,0001**
Nivel IV y V(n=73)	17	23,3	[14,2; 34,7]	**56**	**76,7**	[65,3; 85,8]			
EDACS									
Nivel I a III(n=133)	67	50,4	[41,6; 59,2]	66	49,6	[40,8; 58,4]	0,0002	4,3 [1,9; 9,5]	0,0004
Nivel IV y V(n=47)	9	19,1	[9,1; 33,2]	**38**	**80,9**	[66,8; 90,9]			

Los valores en negrita corresponden a valores de p estadísticamente significativos

GMFCS: Sistema de clasificación de la función motora gruesa. MACS: Sistema de clasificación de las habilidades manuales. CFCS: Sistema de clasificación de las funciones de la comunicación. EDACS: Sistema de clasificación de las habilidades para comer y beber. Nivel I a III corresponden a compromisos leves y moderados, Nivel IV y V corresponden a compromisos severos.

## Discusión

Este estudio caracterizó clínica y funcionalmente a niños, niñas y adolescentes con PC que asisten a centros de rehabilitación de Argentina, haciendo uso de sistemas de clasificación validados y estandarizados. Además, en nuestro conocimiento, es el primer estudio que analiza la asociación entre el compromiso motor y la presencia de discapacidad intelectual y los niveles funcionales en Argentina.

Respecto a la forma clínica de la PC en la población estudiada se observó que predomina la espasticidad, lo cual coincide con referencias internacionales ^8, 9^ . Sin embargo, considerando la distribución topográfica predominó el tipo espástica bilateral, donde ambos lados del cuerpo están afectados e incluye la cuadriplejía y diplejía, por sobre la espástica unilateral y sus otras formas. Esto difiere de lo que se observa en países desarrollados europeos y en Australia, donde se observa una creciente prevalencia de PC espástica unilateral, con una disminución en la prevalencia ^8,10, 11^ . Resulta de interés estudiar las razones de la diferencia en las prevalencias de las formas clínicas, pudiéndose deber a un sub diagnóstico de las tipologías unilaterales de PC en Argentina o que las personas con PC unilateral y posiblemente con compromiso motor más leve asisten a centros de rehabilitación especializados en compromiso motor, aunque no podemos descartar que en nuestro país actúen factores etiológicos o de riesgo diferentes y distintos tanto cualitativa como cuantitativamente o factores aún no identificados o confusores. Por esta razón estamos realizando actualmente un estudio que nos permitirá identificar estos factores en caso de que existieran.

Se observa que, en los centros de rehabilitación de Argentina especializados en discapacidad motora, hay una mayor prevalencia de compromiso motor severo (correspondiente a un nivel V). La muestra de este estudio es consistente en su distribución según niveles de función motora gruesa con la presentada en países de bajos y medios ingresos de Asia y África, y con otros países latinoamericanos, incluyendo Argentina, donde existe una mayor prevalencia de casos más severos ^9, 12–14^ . Por el contrario, en países desarrollados, como Australia y Estados Unidos, los niveles de función motora gruesa leves de PC (Niveles I-II) constituyen el 40 a 60 % de la población, estas diferencias pueden atribuirse principalmente a la incidencia diferencial de los factores de riesgo en gran parte condicionados por el nivel socioeconómico y el acceso inequitativo a servicios de salud oportunos y de calidad, ya que un componente genético influiría en menor medida ^11,15, 16^ .

A medida que se alcanzan niveles más severos de compromiso motor, se observan más probabilidad de presentar mayor severidad en las habilidades funcionales (manuales, en la comunicación y para comer y beber), lo que coincide con lo observado en otros estudios, dada la correlación clínica entre grado de lesión morfológica y afectación funcional neuromotora ^17^ . El nivel de función motora (GMFCS) representa un predictor importante para aquellos aspectos relacionados al funcionamiento y salud física ^18–20^ . Se observó asociación entre la severidad del compromiso motor, y una severidad en las habilidades de comer y beber del EDACS que signifique un peligro de broncoaspiración (EDACS nivel IV) o requiera otra vía de alimentación no oral (EDACS nivel V), esto se vincula con estudios previos donde se establece una asociación moderada entre estos sistemas de clasificación, posiblemente debido a que los centros bulbares que coordinan estas funciones son preservados por los mecanismos compensatorios como ocurre por ejemplo en la asfixia perinatal y afectados con menor frecuencia que otros centros motores ^21^ .

Existe evidencia que los niños, niñas y adolescentes con PC presentan con mayor frecuencia DI ^22–25^ .

La DI como condición asociada de la PC, influye en el nivel funcional global de las personas, aumentando el riesgo de mortalidad ^22^ . De esta forma, aquellas personas con DI presentan más probabilidad de tener mayor compromiso en las funciones motoras, habilidades manuales, de comunicación y de comer y beber. Similares hallazgos se han observado en estudios previos, donde se plantea la necesidad de considerar la DI a la hora de planear los procesos de rehabilitación de cada persona, adaptándolos así a sus necesidades, y adecuándolas a la severidad de las lesiones corticales y de la sustancia blanca subcortical (20,26).

Se encontraron asociación de la DI con mayor severidad en las habilidades para comer y beber, pero es posible que la asociación sea mayor. La disfagia es una problemática difícil de identificar y tratar en las personas con discapacidad, especialmente cuando también presentan discapacidad intelectual. Las dificultades se deben a la presencia de comorbilidades, comportamientos psiquiátricos, dificultades comunicativas, cognitivas y conductuales ^27^ . Es posible también que exista un sub-diagnóstico de disfagia en la población con PC estudiada, sería necesario profundizar en este estudio.

Una limitación del presente estudio fue el hecho de que los datos fueran tomados en centros especializados en discapacidades motoras, al ser instituciones donde existía mayor prevalencia de PC. Esta podría ser un motivo por el que se hallaron más niños, niñas y adolescentes con niveles de compromiso funcional más severos, ya que los menos afectados podrían ser referidos en menor medida a estos centros. Sin embargo, cabe destacar, que los datos presentados en este estudio caracterizan apropiadamente la población de niños, niñas y adolescentes que asisten a centros de rehabilitación de Argentina, y no pretenden comprender a toda la población afectada.

La principal fortaleza de este estudio es que es el primer estudio en caracterizar funcionalmente a niños, niñas y adolescentes con PC que asisten a centros de rehabilitación argentinos. Este estudio se realizó recolectando datos de forma prospectiva en varias provincias, alcanzando una muestra suficiente que permite realizar inferencias con adecuado poder estadístico.

## Conclusión

Se expone que el nivel de compromiso motor y la presencia de discapacidad intelectual influyen en la funcionalidad general de los niños, niñas y adolescentes con PC. La severidad en el compromiso motor y la presencia de discapacidad intelectual se ve reflejada en mayor probabilidad de severidad en habilidades manuales, de comunicación y para comer y beber.
